# Cryptococcoid Sweet Syndrome Following Gastrointestinal Neuroendocrine Tumor Removal: A Case Report

**DOI:** 10.7759/cureus.87483

**Published:** 2025-07-07

**Authors:** Lauren Ashlyn Fletcher, Rithi J Chandy, Taylor R Smith

**Affiliations:** 1 Dermatology, Mercer University School of Medicine, Savannah, USA; 2 Dermatology, AdventHealth Redmond, Rome, USA; 3 Dermatology, Northwest Georgia Dermatology, Rome, USA

**Keywords:** cryptococcoid sweet syndrome, dermatology case report, malignancy-associated sweet syndrome, neutrophilic dermatosis, sweet syndrome

## Abstract

Sweet syndrome, an acute febrile neutrophilic dermatosis, is a reactive disorder that can occur in the setting of infection, malignancy, or medication exposure, although the exact etiology is unknown.Cryptococcoid Sweet syndrome is a rare variant that clinically aligns with Sweet syndrome yet has an initial histologic presentation indicative of a cryptococcal infection. This report describes an 81-year-old man presenting with multiple tender, sharply demarcated, erythematous to violaceous nodules on his forehead, cheeks, and fingers. This eruption occurred 24 hours after he underwent the surgical removal of a neuroendocrine tumor of the ileum. The histopathological examination of a skin biopsy of one of the nodules revealed a predominantly neutrophilic infiltrate present in the superficial dermis with dermal edema and pseudo-*Cryptococcus*-like structures. Periodic acid-Schiff (PAS) stain was negative for fungal organisms. These histologic findings, in light of the clinical presentation, led to a diagnosis of cryptococcoid Sweet syndrome. This report highlights the clinical and histopathological presentation of cryptococcoid Sweet syndrome, a morphologic mimicker of cutaneous cryptococcosis. Taking into consideration the rarity of cryptococcoid Sweet syndrome and the lack of an established relationship between this histologic presentation, malignancy, and surgery, we present this as an interesting case of cryptococcoid Sweet syndrome in the perioperative period of gastrointestinal neuroendocrine tumor manipulation.

## Introduction

Sweet syndrome, an acute febrile neutrophilic dermatosis, is a reactive disorder that can occur in the setting of infection, malignancy, or medication exposure, although the exact etiology is unknown [1,2]. The cryptococcoid variant of Sweet syndrome clinically aligns with classic Sweet syndrome yet has a histologic presentation that mimics a cutaneous cryptococcal infection [2]. Cryptococcoid Sweet syndrome is characterized histologically by cytoplasmic vacuolization and acellular basophilic bodies that resemble the clear capsule and yeast forms of the encapsulated environmental yeast *Cryptococcus*,respectively. Fungal staining is characteristically negative in cryptococcoid Sweet syndrome [1]. This unique histologic variant of Sweet syndrome was first recognized in 2017 [2] and is extremely rare, with only 16 reported cases as of 2024 [1].

## Case presentation

**Figure 1:**
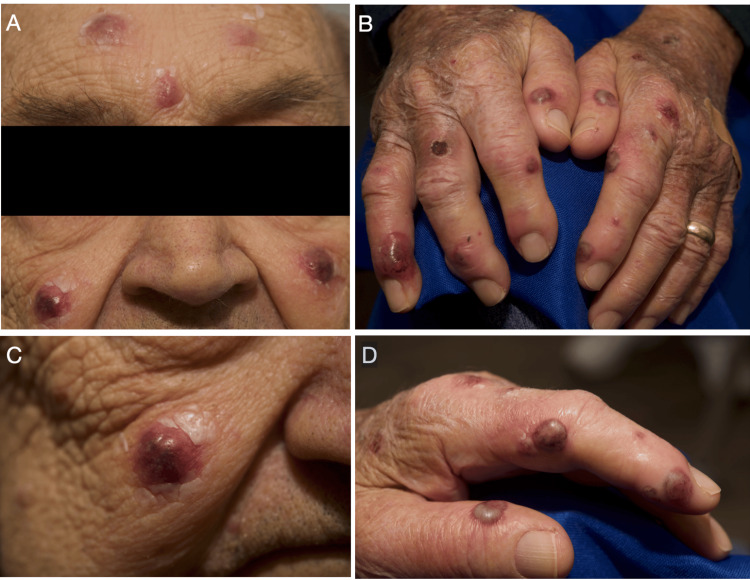
Erythematous to violaceous nodules of the forehead, cheeks, and digits

An 81-year-old afebrile Caucasian man with a past medical history of hypothyroidism, hypertension, and gastroesophageal reflux disease (GERD) presented with a five-day history of an eruption involving the face and digits of both hands. He underwent surgical removal of a neuroendocrine tumor of the ileum 24 hours prior to the onset of the eruption. There were no changes to the patient's medications in the weeks prior to the surgery. The patient reported a similar eruption three years prior that also was not preceded by any new medications. The medical and surgical teams caring for the patient at that time believed the eruption to be an uncontrolled infection, likely osteomyelitis, leading to the amputation of the patient's right fifth finger. The previous eruption self-resolved without further treatment following the amputation of the finger. Notably, the patient has no history of inflammatory bowel disease or hematologic malignancy and has not taken granulocyte colony-stimulating factor. Physical examination revealed multiple tender, sharply demarcated, erythematous to violaceous nodules on his forehead, cheeks, and fingers (Figure 1). Laboratory data showed a mild normocytic anemia with a white blood cell count of 10,340 cells/µL, with neutrophils comprising 85% of the differential. Differential diagnoses considered prior to obtaining a biopsy included Sweet syndrome, given the involvement of both the face and hands, and neutrophilic dermatosis of the hands and erythema elevatum diutinum, which were considered less likely given the facial involvement; infectious causes, such as atypical mycobacterium; and cutaneous metastasis.

**Figure 2:**
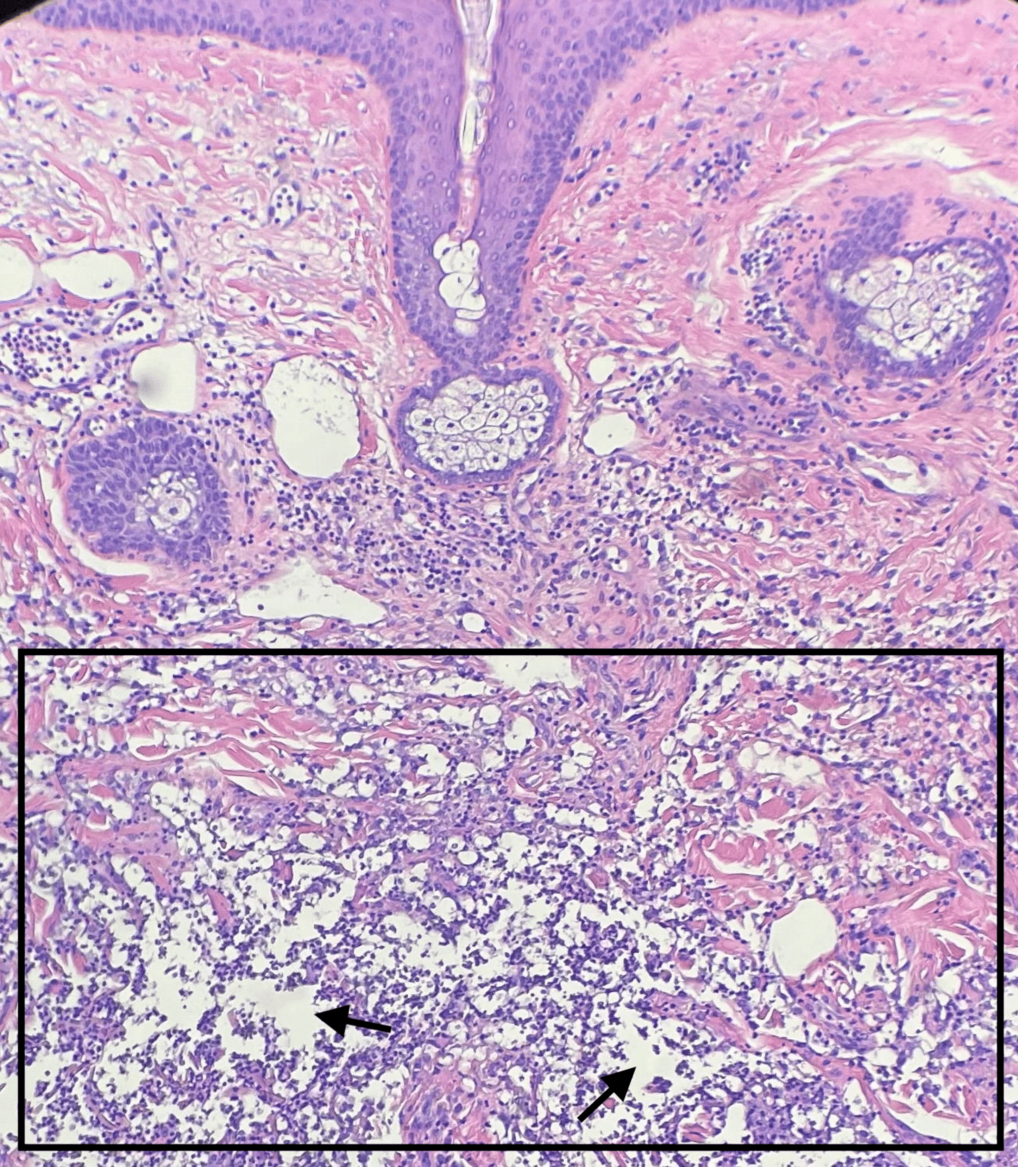
Neutrophilic infiltrate (rectangle) and dermal edema (arrows) in the superficial dermis (H&E, 40× magnification)

**Figure 3:**
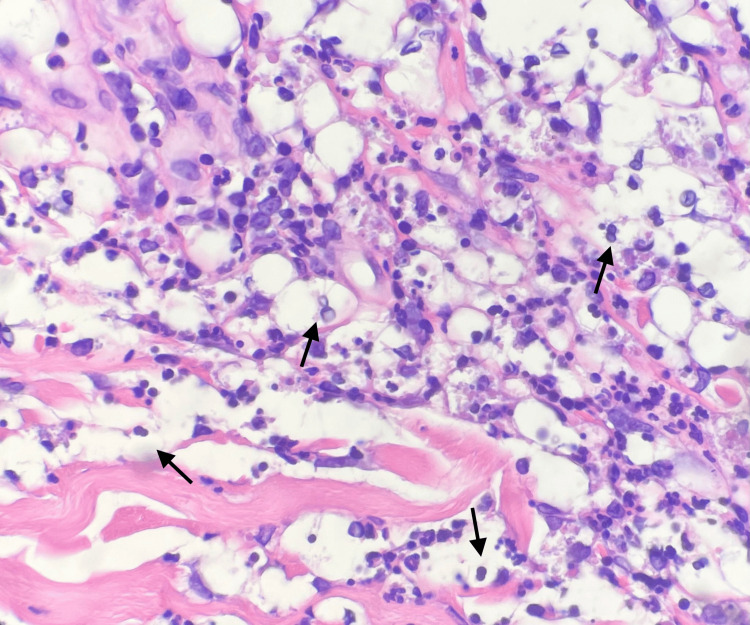
Pseudo-Cryptococcus-like structures (arrows) (H&E, 400× magnification) Cytoplasmic vacuolization and acellular basophilic bodies resembling the clear capsule and yeast forms of *Cryptococcus, *respectively

A deep shave biopsy was performed over an incisional biopsy due to the patient's preference to avoid sutures, given his age and comorbidities. Biopsies of the right frontal scalp and right fourth finger revealed a predominantly neutrophilic infiltrate present in the superficial dermis and dermal edema (Figure 2). Pseudo-*Cryptococcus*-like structures were present (Figure 3). Periodic acid-Schiff (PAS) and mucicarmine stains were negative for fungal organisms. No acid-fast bacilli (AFB) or other bacteria were identified on Ziehl-Neelsen staining for AFB or on Gram-stained sections. These histologic findings, considering the clinical presentation, led to a diagnosis of cryptococcoid Sweet syndrome. The patient had marked resolution of the eruption with a 10-day course of prednisone. Given the patient's gastrointestinal tumor and age, no further workup was conducted, and pathergy testing was not performed, as the eruption fully resolved within two weeks of corticosteroid treatment.

## Discussion

Sweet syndrome is a reactive neutrophilic dermatosis that presents as an acute eruption of tender and erythematous papules, plaques, or nodules [1,2]. The exact pathogenesis of Sweet syndrome is unknown, but it is hypothesized to be an inflammatory response to a trigger such as infection, inflammatory condition, malignancy, or medications. These inflammatory triggers lead to the proliferation of neutrophils that localize to the dermis in response to lymphocytic cytokine signaling [3].

To meet the diagnostic criteria for Sweet syndrome, both major criteria and two of the four minor criteria must be met. The major criteria include the acute onset of tender, erythematous cutaneous papules, plaques, or nodules and the histologic presence of a dermal neutrophilic infiltrate. The minor criteria include fever, leukocytosis, response to corticosteroids, and an association with infection, malignancy, or other underlying conditions [2].

Cryptococcoid Sweet syndrome is a rare variant of Sweet syndrome that was first recognized by Wilson et al. in 2017 [2]. As the name suggests, cryptococcoid Sweet syndrome is a morphologic mimicker of a cutaneous cryptococcal infection. Cryptococcoid Sweet syndrome and cutaneous cryptococcosis are two distinct disease processes that share a name due to their similar histologic findings. These two conditions require vastly different treatments, systemic glucocorticoid therapy for the neutrophilic dermatosis cryptococcoid Sweet syndrome and systemic antifungal therapy for cutaneous *Cryptococcus* infection, emphasizing the importance of an accurate and timely diagnosis [4].

Histologically, cryptococcoid Sweet syndrome is characterized by a dermal inflammatory infiltrate with neutrophils, variable edema of the papillary dermis, and, most notably, vacuolated spaces that resemble the capsule of *Cryptococcus* with basophilic acellular bodies that resemble the budding yeast form of *Cryptococcus* [5]. These histopathologic findings are thought to be caused by autophagic neutrophil degradation with cytosolic vacuolization [6]. Ko et al. used transmission electron microscopy (TEM) to further describe this histologic phenomenon and found that the acellular bodies and capsule-like vacuolated spaces were consistent with remnants of degenerated, autolyzed, apoptosing human cells [7]. The acellular bodies were suggestive of residual degenerated nuclei, and the capsule-like spaces were discovered to be large, voided areas with remnants of the cytoplasm of cells that had undergone autolysis [7]. Additionally, Ko et al. showed on TEM that there was no evidence of fungal cell wall structure or the pathognomonic capsule of *Cryptococcus *[7]. Histochemical fungal stains such as periodic-acid Schiff (PAS) and mucicarmine are characteristically negative [8,9].

While no relationship has been established between malignancy and the cryptococcoid variant of Sweet syndrome, there is an established relationship between malignancy and Sweet syndrome [8]. Hematologic malignancies, specifically acute myeloid leukemia, are most associated with malignancy-associated Sweet syndrome [3,8]. To our knowledge, a direct link has not been established between gastrointestinal neuroendocrine tumors and classic Sweet syndrome or the cryptococcoid variant.

There have been several reports of Sweet syndrome following surgery, with the proposed mechanism being that invasive procedures act as a trigger of inflammation by inducing endothelial damage [10]. Cases of Sweet syndrome have been reported after femoral angioplasty [10], orthopedic surgery [11], varicose vein surgery [12], pneumonectomy [13], coronary artery bypass grafting [14], and partial ileectomy [15]. However, each of these reported cases is of classic Sweet syndrome. To our knowledge, there are no reports of cryptococcoid Sweet syndrome in the postoperative period, and there is no established link between surgery and this specific variant of Sweet syndrome.

This case presents an interesting phenomenon noted histopathologically in the setting of clinically normal-appearing Sweet syndrome. This patient met the major criteria of the acute onset of tender, erythematous cutaneous nodules and a dermal neutrophilic infiltrate on histology. The minor criteria of a response to corticosteroids and an association with malignancy were met, leading to an initial diagnosis of Sweet syndrome. The histologic findings led to an unexpected diagnosis of cryptococcoid Sweet syndrome.

Given the acute onset of cryptococcoid Sweet syndrome 24 hours after the surgical manipulation of a gastrointestinal neuroendocrine tumor, the potential leakage of tumor contents into the bloodstream could be the cause. Additionally, an inflammatory response to surgery should be considered as the cause of this eruption. Drug-induced Sweet syndrome commonly occurs one to two weeks after the initiation of a drug. The patient did not receive any new medications in the weeks prior to surgery. Therefore, drug-induced Sweet syndrome is considered less likely in this case, given the rapid onset of the eruption 24 hours postoperatively. The patient's history of a similar eruption three years prior to the current episode, and before his diagnosis of a neuroendocrine gastrointestinal tumor, raises the question of whether the initial eruption was related to the tumor, as there were no medication changes or other identifiable triggers at that time. Additionally, the unnecessary amputation of the patient's right fifth finger could have been prevented with appropriate diagnosis and treatment, showing the importance of continuing to improve the awareness of this rare condition.

## Conclusions

Taking into consideration the rarity of cryptococcoid Sweet syndrome and the lack of an established relationship between this histologic presentation, malignancy, and surgery, this is an interesting case of perioperative cryptococcoid Sweet syndrome following gastrointestinal neuroendocrine tumor manipulation. This case highlights the need for a high index of suspicion to make a diagnosis of cryptococcoid Sweet syndrome and prompts the consideration of a potential association with malignancy and surgery. Further investigation is necessary.
